# Genome-wide identification and expression analysis of magnesium transporter gene family in grape (*Vitis vinifera*)

**DOI:** 10.1186/s12870-022-03599-5

**Published:** 2022-04-28

**Authors:** Mengqing Ge, Rong Zhong, Ehsan Sadeghnezhad, Abdul Hakeem, Xin Xiao, Peipei Wang, Jinggui Fang

**Affiliations:** College of Horticulture, Nanjing Agricultural University, Nanjing, 210095 China

**Keywords:** MGTs, Grapes, Phylogenetic analysis, Gene expression

## Abstract

**Background:**

Magnesium ion is one of the essential mineral elements for plant growth and development, which participates in a variety of physiological and biochemical processes. Since there is no report on the research of magnesium ion transporter in grape, the study of the structure and function of magnesium ion transporters (MGT) is helpful to understand the dynamic balance mechanism of intracellular magnesium ions and their inter- or intra-cellular activities.

**Result:**

In this study, we identified the members of MGT protein family in grape and performed the phylogenetic and expression analysis. We have identified nine *VvMGT* genes in grape genome, which are distributed on eight different chromosomes. Phylogenetic analysis showed that MGT family members of grapes were divided into five subfamilies and had obvious homology with *Arabidopsis*, maize, and pear. Based on transcriptome data from the web databases, we analyzed the expression patterns of *VvMGTs* at different development stages and in response to abiotic stresses including waterlogging, drought, salinity, and copper. Using qRT-PCR method, we tested the expression of grape *VvMGTs* under magnesium and aluminum treatments and found significant changes in *VvMGTs* expression. In addition, four of the MGT proteins in grape were located in the nucleus.

**Conclusion:**

Overall, in this study we investigated the structural characteristics, evolution pattern, and expression analysis of *VvMGTs* in depth, which laid the foundation for further revealing the function of *VvMGT* genes in grape.

**Supplementary Information:**

The online version contains supplementary material available at 10.1186/s12870-022-03599-5.

## Background

Magnesium ion is the most abundant divalent cation in plant cells, which plays an important role in plant growth and development [1, 2]. As a coenzyme of ATP, a magnesium ion is the activator of many enzymes, especially phosphorylases and kinases [3, 4]. Magnesium ions can stabilize chromatin and DNA structure and regulate the synthesis of ribosomes. Furthermore, it is an indispensable element in the process of DNA replication, RNA transcription, and translation [5]. Magnesium ion participates in the establishment of transmembrane electron gradient [6], maintains intracellular osmotic pressure, and regulates the activity of various intracellular enzymes [7]. As an important component of chlorophyll, magnesium ion participates in plant photosynthesis [8, 9]. Recent studies demonstrated that magnesium ions also play an important role in reducing the toxicity of aluminum salt in plants [10–12]. Therefore, the transport of magnesium ions in the plasma membrane can activate the different mechanisms inside and outside of the cell.

In total, five families of magnesium ion transporters discovered in bacteria, fungi, animals, and higher plants, which included CorA class, Mg^2+^ / H^+^ exchanger, ion channel, P-type phosphatase, and MgtE gene family [13–17]. Among them, first, the CorA protein family was determined as magnesium ion transporter and also deeply studied [4]. There are two hydrophobic transmembrane regions in the C-terminal of these proteins, which are important regions for the transmembrane transport of magnesium ions [18]. There is a completely conserved GMN (Gly-Met-Asn) tripeptide motif near the end of the first transmembrane region [4]. Mutation analysis showed that CorA protein lost the function of magnesium ion transport when an amino acid mutation occurred on GMN motif [19].

In plants, the MGT protein family have been identified in *Arabidopsis*, Rice, Maize, Dendrobium officinale, Brazilian rubber, and Pear [13, 14, 16, 17, 19, 20]. Ten *AtMGTs* genes were identified in *Arabidopsis* [16] and were divided into five subfamilies according to the gene structure and evolutionary relationship. Meanwhile, MGT protein family are homologous to CorA proteins [21]. The protein encoded by *AtMGTs* gene was located on the cell membrane, vacuole membrane, chloroplast membrane, and mitochondrial membrane [15, 16]. *AtMGT1* is involved in the absorption of magnesium ions by roots [16]; although, *AtMGT4*, *AtMGT5,* and *AtMGT9* play a role in pollen development in *Arabidopsis* [22, 23]. Furthermore, *AtMGT6* and *AtMGT7* are involved in resistance to low magnesium stress in *Arabidopsis* [23, 24]; while *AtMGT2* and *AtMGT3* can be involved in the regulation of magnesium ion in mesophyll cells [25–28]. *AtMGT10* plays an important role in the magnesium ion transport system of chloroplast [15]. In rice, *OsMGT1* was identified as a magnesium transporter gene, which participates in the absorption of magnesium ions by roots under aluminum stress, thereby enhancing the resistance to aluminum stress [13]. Subsequently, Saito et al. analyzed the expression and function of all members of the gene family and identified nine *OsMGTs* genes in rice [19]. Li et al. also identified twelve *ZmMGTs* in maize genome, but only 5 members had the ability to transport magnesium ions [29]. It was proved that *ZmMGT12* with a circadian rhythm pattern was able to transport magnesium iron and its expression can be induced by light [30]. And *ZmMGT10* was specifically expressed in maize roots [31]. Zhang et al. identified *DoMGT1* in *Dendrobium officinale* [32], which had tissue-specific expression and only expressed in root, stem, and leaf. Yang et al. cloned the *HbMGT10* gene in *Hevea brasiliensis* and found that it functions magnesium ion transport [33]. It is highly expressed in leaves and participates in the transport of magnesium ions across the chloroplast membrane of rubber leaves. Zhao et al. identified sixteen *PbrMGTs* in Pear and verified the function of *PbrMGT7*, which *PbrMGT7* is involved in the regulation of magnesium ion transport between cytoplasm and mitochondria [34, 35]. And it also participates in the regulation of dynamic balance of magnesium ion in pollen tube growth [35, 36]. *MGT* genes were also identified in *Solanum lycopersicum* and *Brassica napus* [17, 20]. With the publication of genome-wide sequencing data of more plants, more and more MGT gene families will be identified and discovered.

Magnesium ion transporters play an important role in the absorption, transport, and maintenance of magnesium homeostasis [2, 8]. Studies on *Arabidopsis*, rice, maize, and other plants have shown that members of the MGTs family are involved in regulating various activities of biological processes. However, no related research on *MGT* genes has been seen in grapes. In recent years, the phenomenon of magnesium deficiency in grapes in southern China’s vineyards has become more and more serious, and the lack of magnesium nutrition in grapes led to poor growth and development of grapes and reduced fruit quality [37]. In order to solve the above problems, it is necessary to conduct a bioinformatics analysis of the grape magnesium ion transporter gene family and understand the mechanism of magnesium ion transport and distribution.

In this study, we used bioinformatics methods to identify the MGT gene family members and analyze its development process based on the grape genome data. Furthermore, the gene structure, promoter sequence, and physicochemical properties of the protein were also analyzed. In addition, we also studied the expression of *VvMGTs* in different tissues and organs in response to abiotic stresses. These comprehensive results help us to further understand the potential role of Mg^2+^ transporters in grape and solve the problems related to magnesium nutrition in grape cultivation and production.

## Result

### Identification of MGTs in grape

**Fig. 1:**
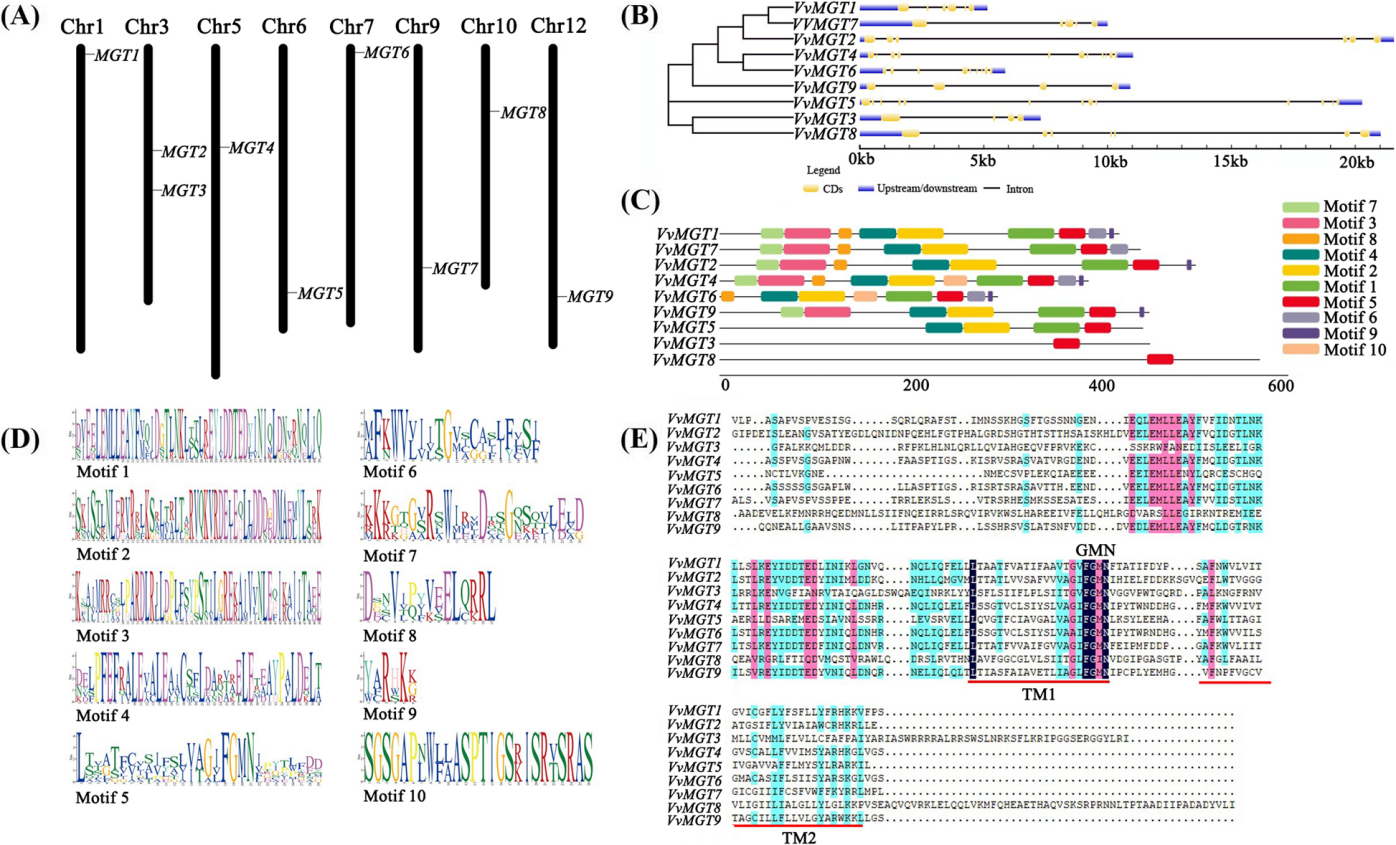
Chromosome location, phylogenetic tree, structural gene features and conserved protein motif of *MGT* genes in grape. **A** Chromosomal location of nine *VvMGT* genes in grape. **B** Structure and phylogenetic analysis of *VvMGT* genes. The blue boxes, black lines and yellow boxes in the gene structure diagram represent untranslated regions(UTRs), introns and coding sequence (CDS), respectively. Gene models are drawn to scale as indicated on the *x*-axis. **C** Conserved motif in MGT proteins. Ten motifs (motif1 to motif 10) were identified with MEME tool and representation of each motif was illustrated with a different color. The lengths and positions of the colored blocks correspond to the lengths and positions of the motifs in the individual protein sequence, respectively. **D** MEME-identified sequence motifs present in the protein sequence of *VvMGT* genes. **E** Multiple sequence alignment of *VvMGT* genes showing their conserved regions and GMN motif, similar amino acids are highlighted in same color, respectively. Predicted C-terminal transmembrane domains (TM1 and TM2) and the conserved GMN motif are indicated

We searched and downloaded the Hidden Markov model file of MGT gene family through Pfam protein family database (Pfam number: PF01544). Based on the V2.1 Grape Genome Database, nine genes of magnesium ion transporter were identified and located on the eight chromosomes (Chr1, 3, 5, 6, 7, 9, 10, and 12) (Table 1). *VvMGT1* and *VvMGT6* were located near the top of different chromosomes and *VvMGT4* and *VvMGT8* were distributed in the upper part of chromosome. Meanwhile *VvMGT2* and *VvMGT3* were located in the middle of chromosome 3 but *VvMGT5*, *VvMGT7* and *VvMGT9* were located in the lower part of different chromosomes (Fig. 1A). The length of *VvMGT1*, *VvMGT3,* and *VvMGT6* was 5153 to 7300 bp, while the other genes were longer, ranging from 10,002 to 21,561 bp. However, there was no significant difference in the length of translation coding region, the length of CDS was between 882 to 1713 bp. Molecular analysis of the full-length deduced polypeptides indicated that the putative proteins of these *VvMGT* genes contain 293 to 570 amino acids (predicted 32.01 to 64.65 kDa in molecular weight) with their *p*I calculated ranging from 4.57 to 8.92. According to web-based prediction of *VvMGTs* location in cells, different members of the MGT gene family were found in the cell membrane, cytoplasm, nucleus, endoplasmic reticulum, chloroplast, mitochondria, golgi, vacuole, and catalase.

**Table 1:** Characteristics of MGT family members in grape

Gene name	Gene ID	Gene size (bp)	CDS (bp)	Number of amino acids(aa)	MW (kDa)	*p*I	Protein formula	Chr location	Subcellular localization
*VvMGT1*	VIT_201s0011g00450	5153	1266	421	47,449.35	5.42	C_2115_H_3341_N_567_O_633_S_19_	Chr1:453195–458,647	Nucleus
*VvMGT2*	VIT_203s0091g01190	21,561	1509	502	55,485.66	4.95	C_2426_H_3869_N_683_O_767_S_19_	Chr3:7831679–7,853,239	Chloroplast, Nucleus
*VvMGT3*	VIT_203s0097g00520	7300	1365	454	51,460.32	8.92	C_2308_H_3651_N_653_O_650_S_16_	Chr3:10861262–10,868,561	Chloroplast
*VvMGT4*	VIT_205s0020g04720	10,359	1170	389	43,470.61	4.97	C_1921_H_3067_N_525_O_590_S_16_	Chr5:6575685–6,586,713	Chloroplast
*VvMGT5*	VIT_206s0061g01060	20,176	1341	446	49,799.66	6.18	C_2201_H_3574_N_610_O_655_S_23_	Chr6:18720855–18,741,404	Nucles
*VvMGT6*	VIT_207s0141g00590	5868	882	293	32,901.06	4.57	C_1441_H_2292_N_392_O_465_S_11_	Chr7:335891–341,775	Nucles
*VvMGT7*	VIT_209s0018g00600	10,002	1335	444	50,492.17	5.03	C_2251_H_3590_N_604_O_669_S_21_	Chr9:16816304–16,826,350	Nucles
*VvMGT8*	VIT_210s0003g02750	21,027	1713	570	64,365.38	6.59	C_2858_H_4528_N_826_O_837_S_16_	Chr10:4824556–4,845,582	Chloroplast
*VvMGT9*	VIT_212s0034g02550	10,920	1362	453	50,457.91	5.47	C_2238_H_3584_N_620_O_669_S_18_	Chr12:19013210–19,024,129	Nucleus

### Structural cluster analysis of *VvMGTs* genes

The clustering of gene families and the analysis of gene structure can provide important clues for revealing the evolution of gene families. We used MEGA7.0 software to analyze nine *VvMGTs* genes (Fig. 1B), which is divided into five categories (Class I, II, III, IV, and V). We found that *VvMGT1*, *VvMGT7,* and *VvMGT2* belonged to class I, *VvMGT4* and *VvMGT6* belonged to class II, *VvMGT3* and *VvMGT8* belonged to class V, and finally *VvMGT5* and *VvMGT9* belonged to classes III and IV respectively. Using the full-length gene sequences and CDS sequences of all genes, the *VvMGT* genes structure was analyzed on the GSDS. The results showed that all gene sequences of the MGT gene family have untranslated regions at both 3’ends and 5’end. The number of exons is between four to thirteen, the length is relatively short, and the intron sequence is long. Combined with gene cluster analysis, it found that the number of exons and introns in the same category are relatively close.

### Analysis of conservative domains

The conserved domain of *VvMGTs* genes was identified by MEME. The specific information is shown in Fig. 1C, D. We found that the types of clustered motifs among a group of genes were basically similar, and the arrangement of motifs was also very similar. For example, the number of motifs in *VvMGT1*, *VvMGT2,* and *VvMGT7* was ranged from eight to nine. *VvMGT3* and *VvMGT8* have only one motif5. The most frequently occurring of all members is motif5 and amino acid sequence contains Gly-Met-Asn, which is a conserved motif of the transmembrane region of the MGT family. Through DNAMAN comparison analysis, it was found that the grape MGT gene family contains two typical transmembrane regions (Fig. 1E). Among them, the GMN motif in the *VvMGT8* sequence was mutated, and methionine replaced with isoleucine, forming a variant GIN motif.

In order to investigate the sequence similarity between *VvMGTs* genes, we used the EMBOSS website to compare the nucleotide and amino acid sequences of grape MGT family members (Table 2). Sequence comparison revealed that the nucleotide sequence homology of these genes in the coding region is 34.7 to 60.2%, while the amino acid sequence homology is 16.7 to 68.60%, and there are only nine gene pairs above 50%. Although the sequence homology of the MGT gene family is not high, all members have GMN marker sequences.

**Table 2:**
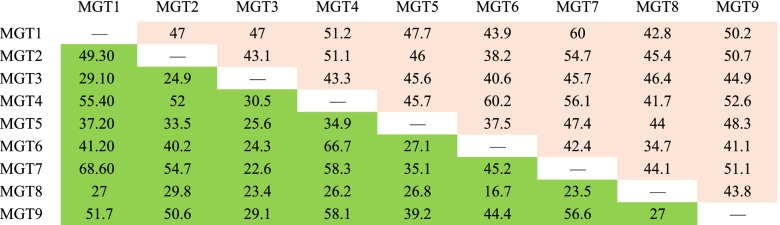
Coding reging nucleotide and amino acid sequence pairwise comparisons(% similarty) between grape MGT gene family

### Protein structure analysis of MGT gene family members

The secondary structure of a protein mainly refers to how the protein itself folds and coils. We found (Table 3) that there are mainly four elements in the secondary structure of the protein in grape MGT gene family including *α*-helix, *β*-turn, extended chain, and random coil. Except for *VvMGT2*, *α*-helix has the highest proportion among other family members, followed by random coils and extended chains, and the least proportion is *β*-turn. The proportion of *α*-helix in each member is about 50%, of which the highest is 59.39% and the lowest is 42.03%. Meanwhile, the proportion of random coils is about 35%, of which *VvMGT2* has the highest proportion of irregular curling, up to 45.22%. Furthermore, the proportion of extension chains in each member is about 10%, of which the highest accounts for 15.00% and the lowest reaches 8.33%. The *β*-turn with the smallest proportion among the 4 elements accounts for 1.90–5.29% of each member.

**Table 3:** Secondary structure of nine *VvMGT* proteins

	H(%)	T(%)	E(%)	RC(%)
*VvMGT1*	54.16	1.90	9.50	34.44
*VvMGT2*	42.03	3.59	9.16	45.22
*VvMGT3*	46.92	5.29	12.56	35.24
*VvMGT4*	54.24	3.08	9.77	32.90
*VvMGT5*	51.79	3.14	9.64	35.43
*VvMGT6*	59.39	2.39	6.83	31.40
*VvMGT7*	51.13	3.15	8.33	37.39
*VvMGT8*	45.44	3.86	15.00	35.61
*VvMGT9*	50.77	2.21	10.38	36.64

*H* Alpha helix (*α*-helix), *T* Beta turn (*β*-turn), *E* Extended strand, *R* Random coil

**Fig. 2:**
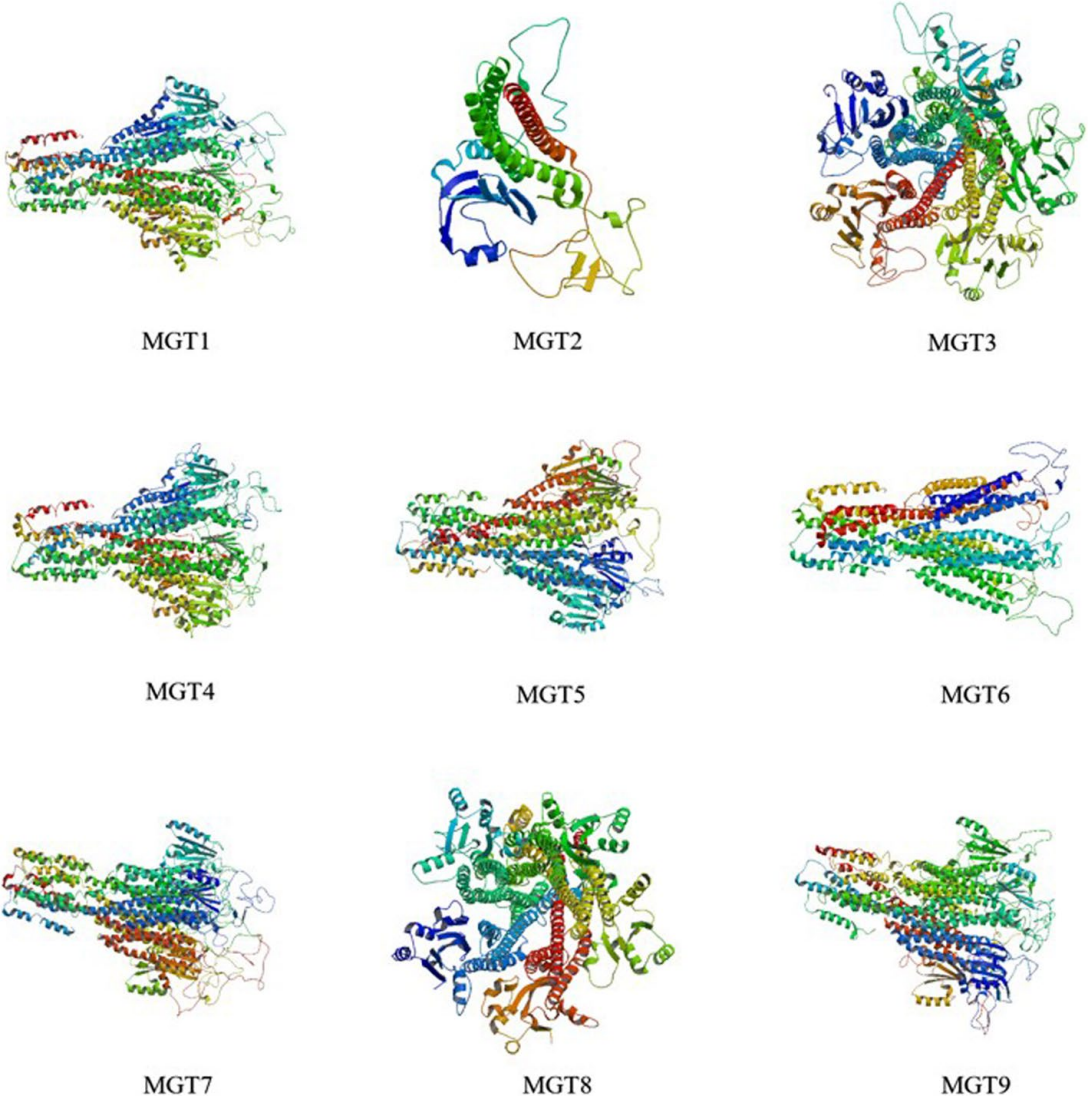
Predicted tertiary structure of nine MGT protein in grape. The protein structures all have the same domain color schemes. Structures reveal a high degree of structural homology in most gene members

As shown in Fig. 2, the protein tertiary structure related *VvMGT*s was constructed by Swiss-Model homology modeling. It can be seen that the protein structures of the same group of members are similar to each other. For example, *VvMGT3* and *VvMGT8* belong to the same group with a similar structure. Besides, the number and types of secondary elements between members of *VvMGTs* including *VvMGT1*, *VvMGT4*, *VvMGT5*, *VvMGT6*, *VvMGT7,* and *VvMGT9* are not much different and made a similarity in their tertiary structures. Noticeably, *VvMGT2*, its protein tertiary structure is different from other family members, and there may be differences in function with other members.

### Analysis of cis-elements on promoter sequences of *VvMGTs*

**Fig. 3:**
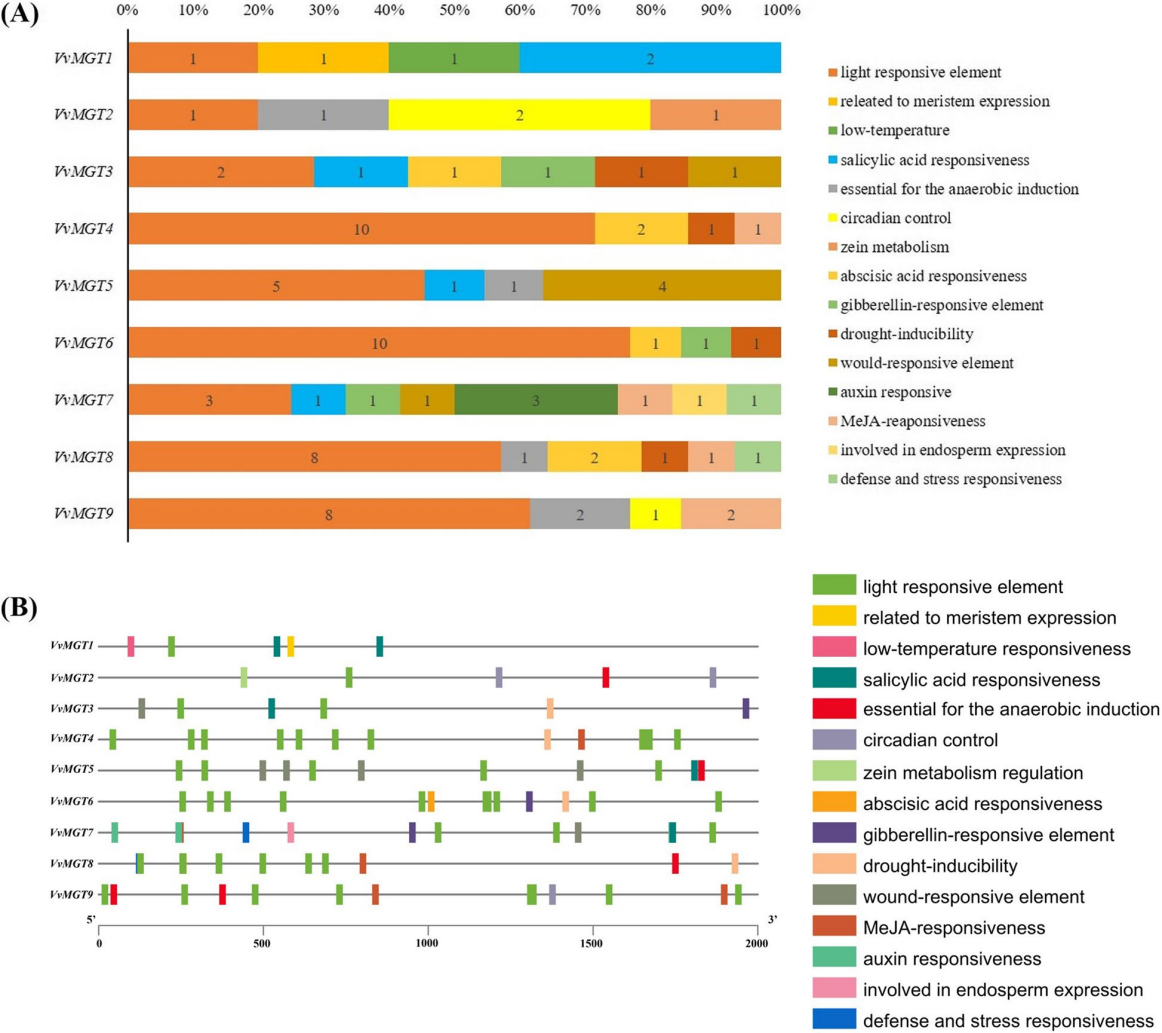
Comparism of *VvMGT* genes promoter sequence. **A** Number and percentage of *cis*-elements occurrence in 9 *VvMGTs* of upstream sequence in grape. The number in bars indicates the number of *cis*-element. The *x*-axis of the upper histogram indicates the percentage frequency of each *cis*-element. **B** Distribution of *cis*-elements on the promoter of *VvMGTs*. Different color bars represent different *cis*-elements. The *x*-axis indicates the length of promoter sequence

In order to understand the gene function and transcriptional regulation mechanism of *VvMGTs*, we obtained 2000 bp upstream of the start site of each gene from the Grape Genome Database and analyzed the *cis*-elements in the promoter regions. In addition to the core components CAAT-box and TATA-box, we found a total of fifteen *cis*- regulatory elements that respond to hormones, light, circadian, biotic and abiotic stresses. Among them, the promoter region of each gene contains a large number of light-responsive elements, accounting for more than 20%. The types of cis-elements in promoter region of *VvMGT* are different (Fig. 3A). For example, *VvMGT7* has the most *cis*-element types in promoter region. *VvMGT3* and *VvMGT8* contain six different kinds of cis-elements; while *VvMGT1, VvMGT2, VvMGT4, VvMGT5, VvMGT6,* and *VvMGT9* also have four different kinds of *cis*-elements. In addition, the promoter sequence of each gene has a different number of each type of *cis*-element.

We also used TBtools software to make the distribution map of promoter sequence according to the number and positions of *cis*-elements (Fig. 3B). The total number of *cis*-elements in *VvMGTs* promoter region was ranged from five to fourteen. The promoter regions of *VvMGT4* and *VvMGT8* have a high content of *cis*-elements, each containing 14 *cis*-elements, while *VvMGT1* and *VvMGT2* contain only five *cis*-elements. In addition, the promoter distribution map shows that almost all the *cis*-elements are distributed throughout the promoter sequence. However, the *cis*-elements of *VvMGT1* are near to start position of promoter sequences.

### Phylogenetic analysis of MGT protein

**Fig. 4:**
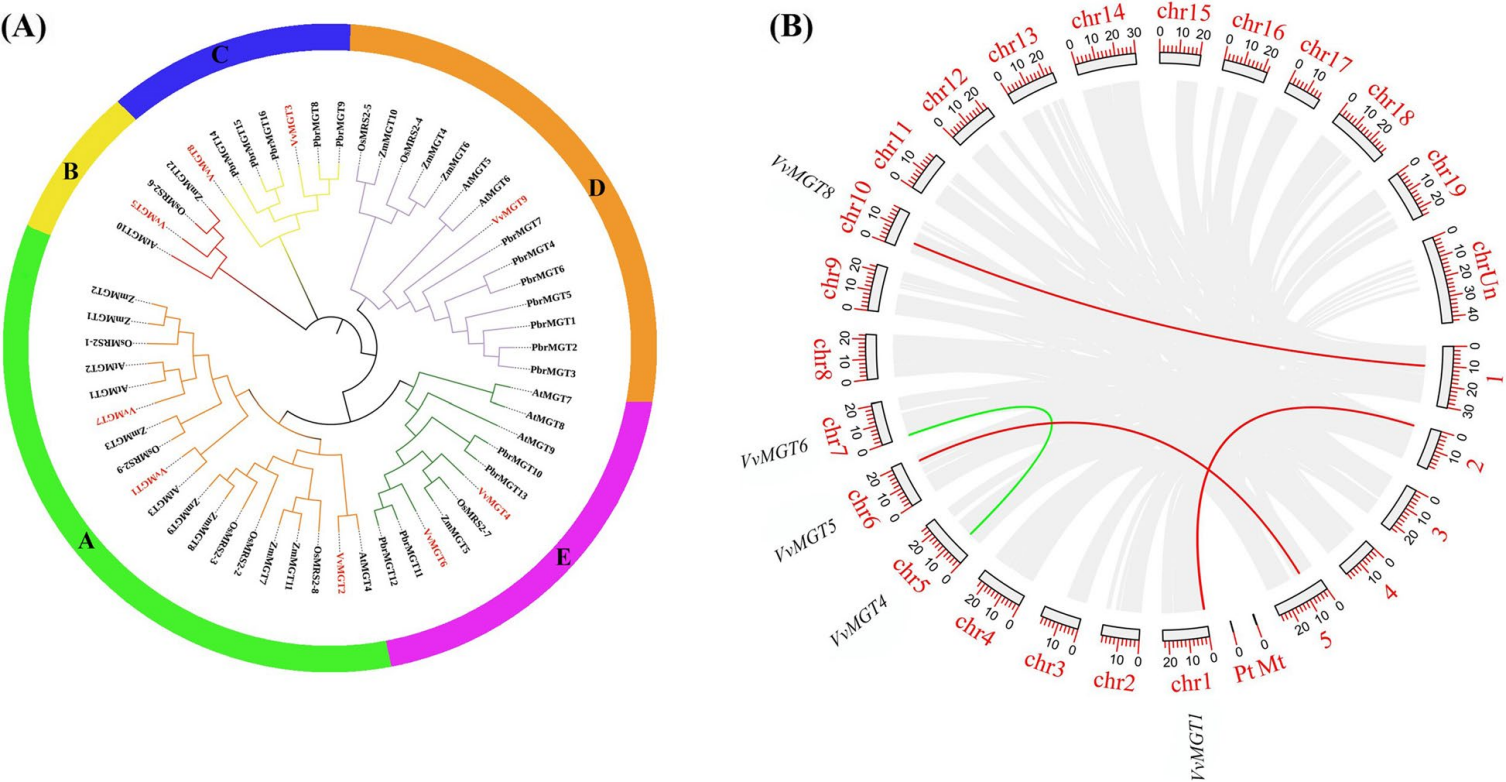
Evolutionary analysis of *VvMGTs*. **A** Phylogenetic tree constructed using 56 full-length MGT proteins from *Arabidopsis*, rice, maize, pear and grape. The phylogenetic tree was constructed using the ML method in MEGA7.0 software with 1000 bootstrap replications. All the MGT proteins are divided into five group (A, B, C, D, E). Branch of different colors to clarify subfamily identification easier. **B** Segmental duplication of grape MGT genes and syntenic analysis of grape and *Arabidopsis* MGT genes. The segmental duplicated gene pairs are connected by red lines between *Arabidopsis* and grape, and green line connected segmental duplicated gene pairs in grape

To explore and compare the evolutionary relationship of MGTs in grape with other plants, we constructed a phylogenetic tree based on the maximum likelihood method with fifty-six protein sequences for *Arabidopsis* (10), Rice (9), Maize (12), Pear (16), and Grape (9) (Fig. 4A). Our results indicated five subfamilies with different members including A (19), B (4), C (7), D (15), and E (11). In subfamily A and B, the members of *MGT* genes mainly originated from *Arabidopsis*, Maize, Rice and Grape. In subfamily C, the members mainly belonged to Pear and Grape, while subfamily D and E contain *MGT* genes from five different species. In order to further understand the evolutionary behavior of grape MGT gene family between species and within species, we analyze gene collinearity in grape and *Arabidopsis* to figure out the expansion process of MGT family members (See Fig. 4B). Some genes in the *Arabidopsis* genome have a collinear relationship with the grape *MGT* genes, such as *VvMGT1*, *VvMGT5*, and *VvMGT8*. In addition, we found that there is no tandem replication event in the grape MGT family, only one fragment repeat, *VvMGT4* and *VvMGT6*.

### Expression analysis of *VvMGT* genes in different organs and tissues

**Fig. 5:**
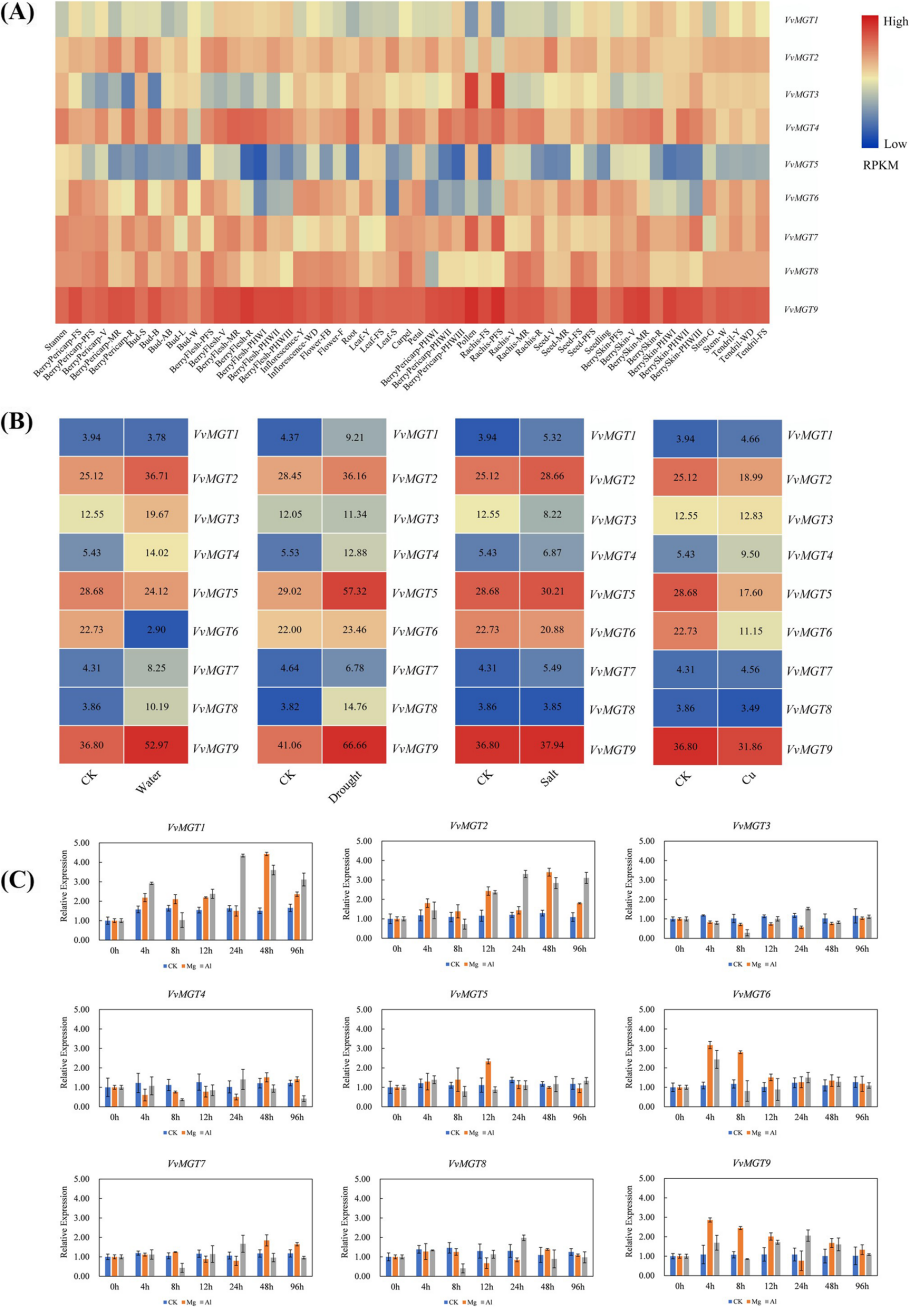
Expression pattern of *VvMGT* genes in grape. **A** Expression profiles of *VvMGT* genes in different grape organs, tissues and developmental stages. **B** Expression profiles of genes in response to water, drought, salt and Cu stresses. **C** The grapevine leaves were collected at 0, 4, 8, 12, 24, 48, and 96 h after treatment and gene expression was analysed by qRT-PCR The data are expressed as mean ± SD (*n* = 3)

In order to obtain more information related to *MGT* genes, we used the transcriptome data of GEO database (GSE36128) containing the expression data of fifty-four tissues or organs during grape development. We used TBtools software to map the expression of *VvMGTs* genes in different developmental stages of grape and different organs (Fig. 5A). The results showed that members of the same subfamily differently expressed in grapes. For example, *VvMGT1*, *VvMGT2*, and *VvMGT7* belonged to the same subfamily, however, the expression level of *VvMGT1* during the entire development process is lower than others. *VvMGT1* and *VvMGT2* were lowly expressed in Pollen and Rachis-PFS but the expression of *VvMGT7* was high in both. This indicates that the expression of *MGTs* genes alters in different tissues during the development of grapes and is dependent on the characteristics of tissue specificity and spatiotemporal. For example, *VvMGT3* is only highly expressed in Pollen and Rachis-PFS and may participate in the signal transduction process of pollen and peel during growth and development. The expression of *VvMGT6* was low in leaf senescence and fruit. According to the gene expression profile, *VvMGT9* was highly expressed in all tissues and organs durin g the whole growth and development period, although, the expression of *VvMGT5* was low in different tissues at different growth stages.

### Expression of *VvMGTs* gene under abiotic stress

There are four common abiotic stress including waterlogging, drought, salinity, and copper in vineyards, which *VvMGTs* expression can be involved in different pathways of resistance or adaptation. Here, we used transcriptome data related to *VvMGTs* to analyze the changes in gene expression under abiotic stresses (Fig. 5B). Under waterlogging stress, *VvMGT6* expression was significantly down regulated. Meanwhile, the expression of *VvMGT2*, *VvMGT5*, *VvMGT6,* and *VvMGT9* was higher in response to salt stress but the expression of *VvMGT6* was down regulated under salinity. Under copper stress, *VvMGT2*, *VvMGT5*, *VvMGT6*, *VvMGT8,* and *VvMGT9* were down regulated. According to the gene expression profile in Fig. 5B, we also found that *VvMGT9* was highly expressed under three different abiotic stresses including waterlogging, drought, and salt stress.

We also performed in vivo experiment under 2% MgSO_4_ and 0.2%AlCl_3_ solution that was sprayed on grape seedlings and the expression of *VvMGTs* genes was detected by qRT-PCR (Fig. 5C). After magnesium treatment, the expression level of *VvMGT1*, *VvMGT6*, and *VvMGT9* increased within 4 till 8 h than 0 h, while the expression of *VvMGT2* and *VvMGT3* decreased and reached at the lowest level after 24 h, and then gradually returned to normal expression level. After 96 h of magnesium treatment, the expression levels of *VvMGT2*, *VvMGT3*, *VvMGT5*, *VvMGT6*, *VvMGT8* and *VvMGT9* returned to normal levels. Under aluminum treatment, the gene expression of *VvMGT1*, *VvMGT2*, *VvMGT4*, *VvMGT5*, *VvMGT6*, *VvMGT7*, *VvMGT8* and *VvMGT9* increased after 4 h, decreased after 8 h, and then gradually increased during 24 h. These genes decreased slowly and tended to be flat after 24 h. Meanwhile, *VvMGT1* and *VvMGT2* genes remained at a high level after 96 h of aluminum ion treatment but the expression level of other members was similar to 0 h.

### Subcellular localization of VvMGT proteins in grape

**Fig. 6:**
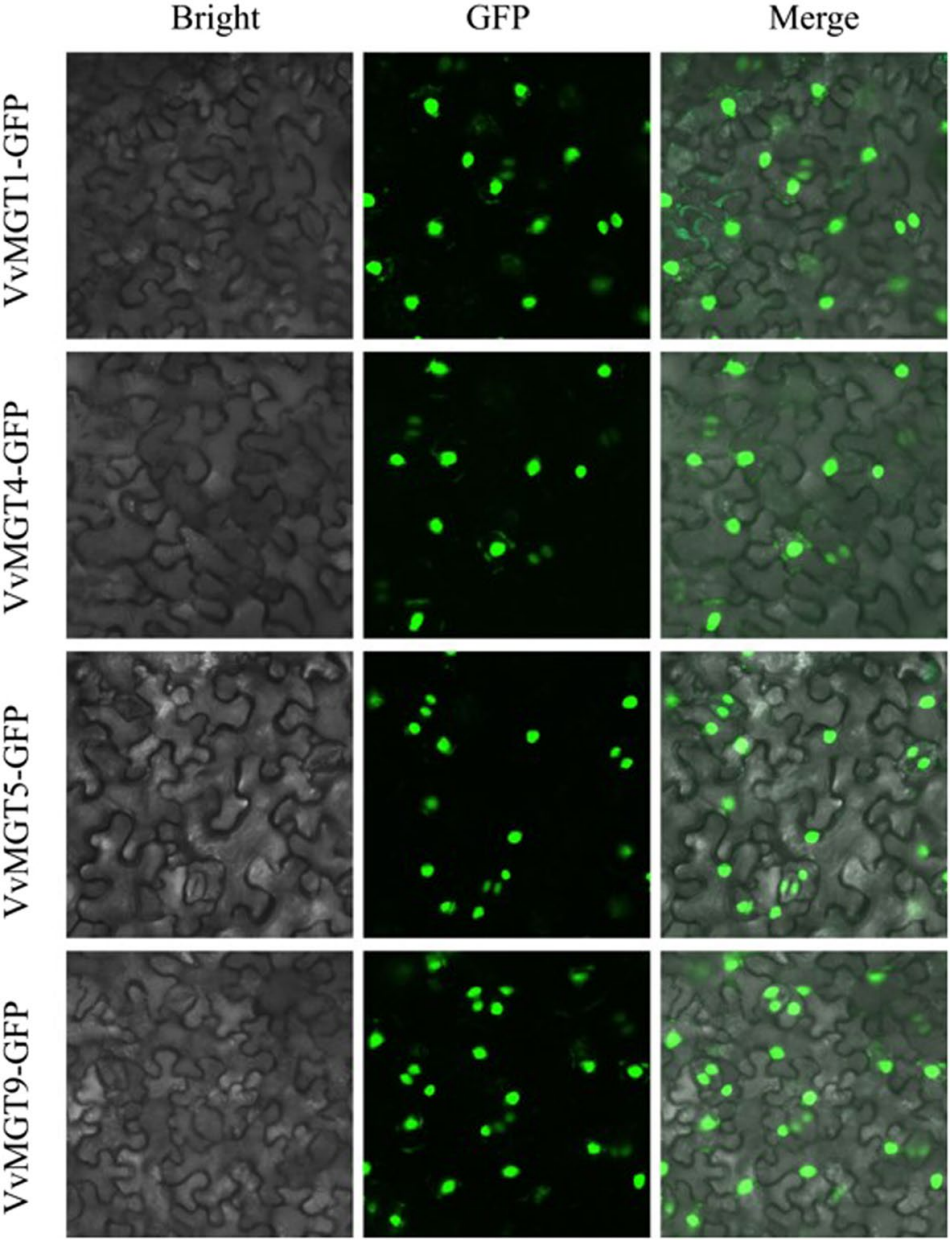
Subcellular localization of four MGT proteins. Four VvMGT*-*GFP fision proteins were expressed transiently in tobacco leaves in an independent manner, and the results were visualized by laser confocal microscopy. The brightfield images are shown in the first panels. The green fluorescence channel are shown in the second panel. The third are the merged images. Bar = 20um

To further investigate the function of *VvMGT* genes in grape, four *VvMGT* genes were chosen as candidate genes with which to explore subcellular localization. Based on the presence of targeted sequences in the protein, the predictive result from plant-mPLoc website showed that most of the MGT proteins were predicted to be located in the nucleus and chloroplast (Table 1). Four MGT genes from different subfamilies were selected to test the results of subcellular localization prediction experimentally, using transient expression assays of fusion proteins between the MGT and the reporter green fluorescent protein (GFP). The recombinant plasmid of 35 s::*VvMGT*-GFP was transformed into *Nicotiana benthamiana* leaves by the Agrobacterium-mediated method. The results showed that the experiment data are basically identical with the plant-mPLoc website predicted ones. Using laser confocal microscopy, for tobacco cells transformed with the recombinant plasmids, however, the green fluorescence of *VvMGT1-GFP, VvMGT4-GFP, VvMGT5-GFP,* and *VvMGT9-GFP* were observed in the nucleus (Fig. 6).

## Discussion

Magnesium ion is an essential nutrient for plant growth and development. As an important magnesium transporter, magnesium ion transporters play an important role in the biological processes of plants [2, 8]. In this study, we investigated the MGT gene family of grape and analyzed chromosomal localization, gene evolution, protein structure, physical and chemical properties. Furthermore, we performed the gene expression analysis of *VvMGTs* during grape development and various abiotic stresses according to transcriptomics datasets. The detailed analysis of MGT gene family in grape provides important information for understanding its molecular mechanism and possible functions in grape growth and development.

### MGT gene family and its evolutionary analysis in grape

We have identified nine MGT family members in the whole grape genome, which is similar to that in *Arabidopsis* and rice [16, 19]. The length of *VvMGTs* gene sequence and the structure of intron and exon in grape MGT family members were different. The results of gene and protein sequence alignment show that the sequence consistency of MGT family members is not high (Table 3). Also, the *pI* range from 4.57 to 8.92 implies that different MGT proteins might be active in different microenvironments. Although the sequence consistency of the family members is low, GMN motifs were determined and were necessary for magnesium ion recognition in each family member. It seems that the function of *VvMGT8* gene alters with the variation of GMN motif and needs further experimental verification. According to cluster analysis of grape MGT members and other plants including *Arabidopsis*(10), Rice(9), Maize(12) and Pear(16) proved that there are differences in the evolution of *MGT* genes among difference. We also found that *VvMGTs* genes were distributed in five subfamilies. This is consistent with the research results of *Arabidopsis* and rice [16, 19], indicating that the evolution of grape MGT gene family is very conservative. Gene replication is considered to be one of the main drivers of the evolution of the genome and genetic systems. Segment repeats and tandem repeats are the two main reasons for the expansion of plant gene families [38–40]. In this study, the results of collinearity analysis of *VvMGTs* gene in grape genome and *Arabidopsis* genome further illustrated the conservation of *VvMGTs* gene family. The above research results indicate that the grape MGT gene family is a slowly conserved gene family, and fragment duplication is the main driving force for the expansion of members of this gene family. In addition, the analysis of promoter sequences of *VvMGTs* showed that there are many types of *cis*-elements in response to different stimuli, which may be closely related to the multiple functions of *VvMGTs* gene in plant species, especially *VvMGT7* may play a role in different metabolism.

### Functional analysis of MGT genes in grape

To better understand the transport mechanism of *MGTs* in plants, we performed the functional and expression analysis of *MGTs* genes. The expression patterns of ten *AtMGTs* genes in *Arabidopsis* were different in pod, flower, root, stem, and leaf. *AtMGT5* was expressed only in flowers and young pods, while *AtMGT8* gene was not expressed in stem [41]. *AtMGT10* gene is expressed in tender leaves or mature microtubule tissues, which mediates the transport of magnesium ions in chloroplasts and affects chlorophyll metabolism [16, 42]. In rice, nine *OsMGTs* genes were low expressed in undeveloped yellow-green leaves, and expression levels increased in mature leaves [11]. These results indicate that different family members participate in the regulation of plant growth and physiological metabolism through different modes of action. In this study, we found that *VvMGT3* was highly expressed only in pollen and rachis PFS, while *VvMGT6* expression was low in tissue senescence and organs. This indicates that the expression of *VvMGTs* genes in grape growth and development is also tissue-specific and spatiotemporal expression. In addition, the subcellular localization analysis showed that MGT protein in grape may be play a important role in nucleus.

A previous study showed that *AtMGT10* gene is regulated by light and there are binding sites of transcription factors in the promoter region of *AtMGT10*, which it led to a significant increase in the transcriptional level of *AtMGT10* under circadian rhythms [42]. In order to understand more clearly the regulatory network of *VvMGTs* in grape, the *cis*-elements of promoter sequence were analyzed. In *VvMGT2* and *VvMGT9*, we observed the *cis*-element responsive to circadian rhythm, which it suggests the regulation of two genes requires circadian clock-responsive factors in grape. In addition, we found a large number of light- and hormone-responsive elements in the promoter region of *MGTs* gene, as well as drought and low temperature-responsive elements. This suggests that *VvMGTs* play a role in abiotic stress.

In order to investigate the expression of *VvMGTs* genes under environmental stresses, we candidated four abiotic stress including; drought, waterlogging, salinity, and copper damage and analyzed the gene expression patterns using transcriptome data. Under waterlogging, the expression of *VvMGT9* gene was significantly up-regulated, indicating that the gene may be closely related to waterlogging resistance of grape. Under drought stress, *VvMGT5* and *VvMGT9* were significantly up-regulated and the expression level of *VvMGT5* was almost twice as much as that of the control group, indicating that these two genes may play an important role in drought tolerance. The expression of *VvMGTs* also changed under salt and copper stresses but the degree of change was not as obvious as the drought and waterlogging stresses. However, *VvMGT9* highly expressed in waterlogging, drought, and salt stress, we speculated that *VvMGT9* may play an important role in response to abiotic stress of grape. Furthermore, in order to evaluate the expression of *VvMGTs* under ionic stresses, we studied the gene expression changes under magnesium and aluminum in grape. We found that the response mode of *VvMGTs* was different under magnesium and aluminum stresses. Under magnesium treatment, seven genes including *VvMGT1*, *VvMGT2*, *VvMGT3*, *VvMGT4*, *VvMGT7*, *VvMGT8*, and *VvMGT9* reached at the highest level after 24 h. After aluminum exposure, the expression levels of all *VvMGTs* genes were up-regulated in a time-dependent behaviour, except *VvMGT2* that its expression decreased. This suggests that the response mechanism of *VvMGT2* gene in response to aluminum may be different from other members. In conclusion, the expression levels of *VvMGTs* genes are in a time-dependent behavior under magnesium and aluminum ion treatment that play different roles in the regulation of biological processes. Deng et al. showed that overexpression of *AtMGT1* in tobacco could improve the resistance to aluminum toxicity [43]. In *Arabidopsis thaliana*, *AtMGT2* and *AtMGT3* located on vacuole membrane are closely related to the distribution of magnesium and respond to the high concentration of magnesium ion [25, 44]. They can transfer the excessive magnesium ions from the cytoplasm to the vacuole under the condition of magnesium toxicity, reduce the magnesium stress in the cytoplasm, and regulate the dynamic balance of magnesium ions in the cell. *AtMGT6* and *AtMGT10* were highly expressed in magnesium deficient roots [45, 46]. *ZmMGT10* in maize can promote plant growth under magnesium deficiency [29]. The results showed that there were some similarities in function and regulation of MGT gene family members in grape when compared to other species.

It was found that magnesium could alleviate the toxicity of aluminum ion in *Arabidopsis* [47]. Furthermore, Silva et al. found that magnesium can effectively alleviate the aluminum toxicity of soybean roots [48]. MacDiarmid et al. found that under aluminum stress, yeast’s absorption of magnesium was hindered and the growth of cells inhibited [49]. However, after the addition of magnesium, it was found that the yeast recovered the absorption and transport of magnesium. Meanwhile, yeast cells also grew again and the transporter gene in yeast began to express, which alleviated the effect of aluminum toxicity. In our study, the expression of *MGTs* genes in grape was up-regulated under aluminum salt stress, which may be related to the regulation of magnesium ion absorption, transport, and distribution to alleviate aluminum salt stress in grape. The subcellular localization experiments further demonstrated that MGT protein may play an important role in the nucleus.

At present, the transport mechanism of plant magnesium ion is only the tip of the iceberg [12]. There are no systematic studies on the characteristics, expression patterns, and biological functions of magnesium ion transporters in different plants. The molecular mechanism of how magnesium ions are absorbed, transported, unloaded and distributed is unclear. Therefore, it is necessary to study the mechanism of *MGTs* in plants. The identification and expression analysis of *MGTs* genes in grape will lay a foundation for the study of magnesium ion in grape quality improvement and genetic regulation mechanism.

## Conclusion

In this study, we identified nine *VvMGTs* genes based on the genomic dataset of grape for the first time and divided them into five subfamilies. The gene structure, chromosomal localization, conserved domain, protein structure, evolutionary relationship, and functional elements of the promoter region were analyzed. We observed that the expansion of members of the grape MGT gene family is dependent on the fragment duplication as the main driving force and is a slowly conserved gene family. At the same time, the expression profile of *VvMGTs* genes in the whole development period of grape was also analyzed, and the expression of *VvMGTs* gene under abiotic stress was studied. The results indicated that all *VvMGTs* genes could alter their expression in a time-dependent behavior in grape. Furthermore, determination of cis-acting element involved in light, hormone, and environmental stimuli suggest a critical role in the regulation of transcriptional levels of *VvMGT* family memebers for tolerance strategy in grape. Furthermore, the regulation of magnesium ion in grape plays an important role in solving ionic toxicity that can lay a theoretical foundation for the functional research of *VvMGTs.*

## Methods

### Identification of MGT family members and chromosome distribution in grape

To identity a complete list of grapevine *MGT* genes, we downloaded the annotated grapevine proteins from three public databases: the National Centre for Biotechnology Information (https://www.ncbi.nlm.nih.gov), the Grapevine Genome Browser (http://www.genoscope.cns.fr/externe/GenomeBrowser/Vitis/), and the Grape Genome Database (http://genomes.cribi.unipd.it/grape/, V2.1). We used the HMMER (http://hmmer.janelia.org/) Hidden Markov model (PF01544) as a probe to screen all the candidate protein with E-values of less than 2.40e-05. Then, the search results of the protein sequences were further confirmed using SMART (http://smart.embl-heidelberg.de) and Inter ProScan program (http://www.ebi.ac.uk/Tools/pfa/iprscan5/) to ensure their reliability. Finally, all identified *VvMGT* genes were utilized to analyze the amino acid length, molecular weight (MW), and isoelectric point (*p*I) by ExPASY (http://expasy.org). We used the plant-mPLoc website (http://www.csbio.sjtu.edu.cn/bioinf/plant-multi/) for subcellular localization analysis of the *VvMGTs* genes [50]. The map of chromosome distribution was made by MapChart software and named by their chromosomal location [51].

### Analysis of gene structure and protein classification

Based on *VvMGT* genes coding sequence (CDS) and the correspondent full-length gene sequences in NCBI, we used Gene Structure Display Server Software (GSDS.v2.0) (http://gsds.cbi.pku.edu.cn/,2.0) to analyze the structure of the *VvMGT* genes. In order to detect the classification of all *VvMGTs*, the protein sequences of them were determined by Clustal W [52]. The phylogenetic tree was constructed using MEGA7.0 with the Maximum Likelihood (ML) method and the bootstrap test carried out with 1000 replicates [53].

### Conserved motifs analysis and comparative sequence identity of *MGTs* genes

To identify the conserved motif of grape MGT proteins, we submitted the protein sequence of *VvMGTs* to the MEME website (http://meme.nbcr.net/meme) and set the motif number as 10 [54]. We used TBtools software to visualize the results of protein conserved sequences [55]. Two transmembrane (TM) domains and the Gly-Met-Asn (GMN) motifs were marked according to the TMHMM analysis and the sequence alignment using the DNAMAN software. We also used the EMBOSS online website (http://emboss.sourceforge.net/) to analyze the similarity of *VvMGTs* gene and protein sequences.

### Structural prediction and modeling of VvMGT proteins

To identify the structural composition of various Mg^2+^ transporter genes, we use SOPMA (https://npsa-prabi.ibcp.fr/cgi-bin/secpred sopma.pl) prediction protein secondary structure. Then, the protein tertiary structure was constructed by Swiss-Model website (https://swissmodel.expasy.org/) using homology modeling.

### Finding of cis-regulatory elements in promoter regions

We obtained the 2000 bp sequence upstream of the *VvMGT* genes as promoter regions and submitted to the PlantCARE website (http://bioinformatics.psb.ugent.be/webtools/plantcare/html/). Cis- acting elements on the promoter sequences were determined in *VvMGT* genes.

### Phylogenetic analysis

First, we downloaded the protein sequences of grapes, *Arabidopsis*, Rice, Maize, and Pears on the Grape Genome and Phytozome (https://phytozome.jgi.doe.gov/pz/portal.html) websites. Phylogenetic analysis of 56 amino acid sequences of five species was performed using MEGA7.0 software. We used TBtools software to perform collinear analysis of the Grape genome, and *Arabidopsis* genomes.

### Expression analysis of *VvMGT* genes using transcriptome data

The transcriptome data of different organs were downloaded from the Gene Expression Omnibus (http://www.ncbi.nlm.nih.gov/geo/) in different development stages of grape (Date code number: GSE36128).

In order to study the expression profiles of *VvMGTs* in response to various abiotic stresses (Cu, salt, waterlogging, and drought stress), the grapevine transcriptome data in response to waterlogging (SRA accession no. SRP070475) and drought stress (SRA accession no. SRP074162) were retrieved from NCBI database (https://www.ncbi.nlm.nih.gov/sra/SRP070475 and https://www.ncbi.nlm.nih.gov/sra/term=SRP074162, respectively) [56, 57] (Haider et al., 2017; Zhu et al., 2018). Transcriptome data for expression profiles in response to copper (Cu) and salinity were retrieved from published data sets by Guan et al. [58].

The analysis of transcriptome data was based on the Leng et al. method [59], and the RPKM (Reads Per Kilobase per Million mapped reads) values were used to estimate the gene expression level. Expression data was also mapped by TBtools and presented in the heat map format.

### Plant materials and experimental treatment

The two-year-old grape cultivar ‘Kyoho’ was planted in the greenhouse of Baima Teaching Base of Nanjing Agricultural University. We set up one control group and two experimental groups in the test. Control group was sprayed with dionized water and the treatment groups were sprayed with AlCl_3_ and MgSO_4_ solutions. One treatment group was sprayed using 0.2% AlCl_3_ solution, and the other treatment was spraying using 2% MgSO_4_ solution [12, 60, 61]. We collected leaves in a time-course experiment after solution exposure (0, 4, 8, 12, 24, 48, and 96 h). The collected leaves were immediately frozen in liquid nitrogen and stored at − 80 °C for RNA extraction.

### RNA extraction and quantitative real-time PCR (qRT-PCR)

Total RNA was extracted using a kit (Vazyme,Beijing,China) and the reverse-transcribed process performed with a HifairII® 1st Strand cDNA Synthesis SuperMix for qPCR kit (Yeasen,Shanghai,China). All primers were designed by Primer Primer5.0 software for qRT-PCR, which were listed in Table S1. Total volume of reaction mixtures was 10 μL, which included 5 μL of SYBR Green Supermix (Bio-Rad), 2 μL of diluted cDNA, 0.2 μL of each primer, and 2.6 μL double-distilled water. All reactions were performed in three biological replicates. qRT-PCR was carried out using the CFX96 Real-Time PCR Detection system (Bio-Rad, Hercules, CA, USA). The following steps were carried out in PCR: predenaturation at 94 °C for 30 s, followed by 40 cycles of denaturation at 94 °C for 5 s, primer annealing at 60 °C for 15 s, and extension at 72 °C for 10 s. Optical data were acquired after the extension step, and the PCR reactions were subjected to a melting curve analysis beginning from 65 °C to 95 °C at 0.1 °C s^− 1^. *VvUBI* (Gene code LOC100259511) was used as an internal reference gene, and the expression data were calculated by 2^-∆∆CT^ method [62].

### Subcellular localization assays

The coding sequence (CDS) of the genes *VvMGT1*, *VvMGT4*, *VvMGT5* and *VvMGT9* were amplified from ‘Shine Muscat’ grape leaf by PCR, using the corresponding primers, which are listed in Table S1. The amplified PCR products were cloned into the modified pCAMBIA1302-GFP vector carrying the CaMV35s promoter (Clontech, Beijing, China). Subsequently, the fusion plasmids 35 s::VvMGT-GFP were independently transferred into *Agrobacterium tumefaciens* cells (Weidi, Shanghai, China). *Agrobacterium* cells transformed with the respective fusion plasmid were then injected into tobacco (*Nicotiana benthamiana*) leaves, and the green fluorescence signals were visualized with a Zeiss LSM800 Image Browser (ZEISS, Germany) 48 h after transformation. Three independent experiments were performed for each gene.

## Supplementary Information

**Additional file 1.**

## Data Availability

The data presented in this study are available on request from the corresponding author. All databases used in this study are open for public access. The references of these databases are as follow: the Grapevine Genome Browser (http://www.genoscope.cns.fr/externe/GenomeBrowser/Vitis/), the Gene Expression Omnibus (http://www.ncbi.nlm.nih.gov/geo/), the grapevine transcriptome data in response to waterlogging (SRA accession no. SRP070475) and drought stress (SRA accession no. SRP074162) were retrieved from NCBI database (https://www.ncbi.nlm.nih.gov/sra/SRP070475 and https://www.ncbi.nlm.nih.gov/sra/term=SRP074162, respectively).
